# Structural Studies of a Rationally Selected Multi-Drug Resistant HIV-1 Protease Reveal Synergistic Effect of Distal Mutations on Flap Dynamics

**DOI:** 10.1371/journal.pone.0168616

**Published:** 2016-12-16

**Authors:** Johnson Agniswamy, John M. Louis, Julien Roche, Robert W. Harrison, Irene T. Weber

**Affiliations:** 1 Department of Biology, Georgia State University, Atlanta, Georgia, United States of America; 2 Laboratory of Chemical Physics, National Institute of Diabetes and Digestive and Kidney Diseases, National Institutes of Health, DHHS, Bethesda, Maryland, United States of America; 3 Roy J. Carver Department of Biochemistry, Biophysics and Molecular Biology, Iowa State University, Ames, Iowa, United States of America; 4 Department of Computer Science, Georgia State University, Atlanta, Georgia, United States of America; 5 Department of Chemistry, Georgia State University, Atlanta, Georgia, United States of America; University of Pittsburgh, UNITED STATES

## Abstract

We report structural analysis of HIV protease variant PR^S17^ which was rationally selected by machine learning to represent wide classes of highly drug-resistant variants. Crystal structures were solved of PR^S17^ in the inhibitor-free form and in complex with antiviral inhibitor, darunavir. Despite its 17 mutations, PR^S17^ has only one mutation (V82S) in the inhibitor/substrate binding cavity, yet exhibits high resistance to all clinical inhibitors. PR^S17^ has none of the major mutations (I47V, I50V, I54ML, L76V and I84V) associated with darunavir resistance, but has 10,000-fold weaker binding affinity relative to the wild type PR. Comparable binding affinity of 8000-fold weaker than PR is seen for drug resistant mutant PR20, which bears 3 mutations associated with major resistance to darunavir (I47V, I54L and I84V). Inhibitor-free PR^S17^ shows an open flap conformation with a curled tip correlating with G48V flap mutation. NMR studies on inactive PR^S17^_D25N_ unambiguously confirm that the flaps adopt mainly an open conformation in solution very similar to that in the inhibitor-free crystal structure. In PR^S17^, the hinge loop cluster of mutations, E35D, M36I and S37D, contributes to the altered flap dynamics by a mechanism similar to that of PR20. An additional K20R mutation anchors an altered conformation of the hinge loop. Flap mutations M46L and G48V in PR^S17^/DRV complex alter the Phe53 conformation by steric hindrance between the side chains. Unlike the L10F mutation in PR20, L10I in PR^S17^ does not break the inter-subunit ion pair or diminish the dimer stability, consistent with a very low dimer dissociation constant comparable to that of wild type PR. Distal mutations A71V, L90M and I93L propagate alterations to the catalytic site of PR^S17^. PR^S17^ exhibits a molecular mechanism whereby mutations act synergistically to alter the flap dynamics resulting in significantly weaker binding yet maintaining active site contacts with darunavir.

## Introduction

Protease (PR) is essential for the maturation of human immunodeficiency virus (HIV) and is a major drug target for treatment of the HIV/AIDS pandemic [1]. To date, 9 HIV protease inhibitors (PIs) have been approved by FDA for treatment of AIDS [2]. Combination antiretroviral therapy, including nucleoside/non-nucleoside transcriptase, entry, and integrase inhibitors, in addition to PR inhibitors has suppressed viral loads and increased the life expectancy of patients with HIV/AIDS [3–4]. However, the emergence of resistance mutations limits the success of treatment. Although PIs have a high genetic barrier against viral resistance, mutations associated with resistance to each of the 9 approved protease drugs are observed in clinical isolates [5]. Mutations at the substrate binding cleft, called primary mutations, appear early in response to PI therapy and interfere with PI binding. Mutations that appear outside the binding cleft during continuous PI therapy are termed secondary or compensating mutation. Both primary and secondary mutations contribute to resistance to different PIs through several mechanisms [6]. Furthermore, the protease flaps play a critical role in the binding of substrate or inhibitors. Opening of the flaps is necessary for entry of substrate into the binding cleft and flaps in the closed conformation secure the substrate for catalysis. The protease dimer is in dynamic equilibrium between the closed conformation and various open conformational states [7]. Molecular dynamics studies suggest that mutations in the flaps or even in distal regions may affect the flap dynamics and hence the binding of PIs [8–9].

Analysis of multidrug resistant mutants of HIV PR suggests that up to 20 mutations may be necessary to acquire high levels of resistance to several drugs [10]. The mutations act synergistically to evade inhibitors by different mechanisms. One well characterized clinical HIV protease isolate that harbors 6 primary and 7 secondary drug resistance mutations (PR20) is extremely resistant to all FDA approved protease inhibitors [11–14]. PR20 has 3–4 orders of magnitude weaker binding to all clinical inhibitors in comparison to wild type PR [11]. The efficiency of Gag polyprotein processing by PR20 is lower by 4-fold in comparison to PR, although PR20 retains the same order of cleavage as wild type PR [15]. The N-terminal autoprocessing, a prerequisite for stable dimer formation and appearance of mature-like catalytic activity, is not affected by the mutations. Importantly, autoprocessing of the TFR-PR20 precursor expressed in E.coli is not inhibited by darunavir (DRV) and saquinavir (SQV) up to 150 μM relative to the wild type precursor (IC_50_ 1–2 μM) [11]. Structural studies of PR20 show that clusters of mutations produce conformational changes that lower the binding affinity of inhibitors [12–13]. For example, mutations of residues 35–37 in PR20 perturb the flap conformation [13]. Furthermore, the backbone residual dipolar coupling measurement for N-H amide vectors verified that PR20 adopts a wide open conformation in solution unlike the wild type enzyme [16]. Thus, it is important to identify mutational clusters and the molecular basis of extremely resistant variants like PR20. Such knowledge will be beneficial to implement effective therapy and design of novel inhibitors.

Recently, we reported a multidrug resistant HIV protease bearing 17 mutations named PR^S17^. The sequence of PR^S17^ was selected from genotype-phenotype data by a new method for predicting drug resistance using machine learning with a unified encoding of the sequence and 3-D structure [17–18]. This method accurately classified genotype data to predict drug resistance. In addition, it showed excellent correlation between predicted and observed levels of resistance in cross-validated regression analysis [19]. Mean shift clustering with regression analysis identified mutants, such as PR^S17^, with high levels of resistance to multiple drugs that were representative of wide classes of drug-resistant proteins [20]. We demonstrated that purified mature PR^S17^ exhibits extreme resistance to all 8 potent PIs tested [17]. It forms a stable dimer and is ~10- and 2-fold less efficient in processing the Gag polyprotein relative to the wild-type and PR20, respectively [17]. However, PR^S17^ maintains the same cleavage order as PR and PR20. Inhibition of autoprocessing at the N terminus of PR^S17^ of a model precursor (TFR-PR^S17^) by clinical inhibitors is 200 to 800-fold weaker than seen for the mature PR^S17^ [17].

Here, we present the first crystal structures of PR^S17^ in the inhibitor-free form and in complex with DRV. Solution NMR studies were carried out to investigate the open-closed flap conformation of the inhibitor free PR^S17^ bearing an active site D25N mutation. Structures of PR^S17^ are compared with the corresponding structures of PR and PR20. These studies reveal distinct mutational clusters in PR^S17^ and their molecular basis for high drug resistance, and thus provide valuable insights for the design of successful inhibitors targeting highly evolved multi drug resistant proteases.

## Materials and Methods

### Construction, expression and purification of PR^S17^

Synthetic genes encoding the 99 amino acid PR^S17^ and its active site mutant PR^S17^_D25N_ (DNA2.0, Menlo Park, CA) were cloned in pJ414 vector flanked by Nde1 and BamH1 sites, and transformed into *E*.*coli* BL-21 (DE3; Stratagene) [14]. Expression in Luria Bertani medium or in minimal medium for isotope labeling, purification and protein folding were carried out as described previously [21–23].

### NMR methods

The backbone assignment of PR^S17^ was based on 3D TROSY-HNCO and 3D TROSY-HNCACB spectra recorded on an uniformly ^2^H/^15^N/^13^C-enriched (>98%) sample. The steady-state heteronuclear ^15^N-{^1^H} NOE data were collected using TROSY-based ^1^H-^15^N heteronuclear experiments [24]. The ^1^D_NH_ RDCs were derived from the difference in ^1^J_NH_ + ^1^D_NH_ splitting using an ARTSY-HSQC experiment [24] on an isotropic sample and an aligned sample. The alignment of the samples was obtained by the addition of 10 mg/ml squalamine and 5 mM hexanol, yielding a stable ^2^H quadrupole splitting of ~20 Hz. The average experimental error in the measured ^1^D_NH_ RDCs is 0.15 Hz. All NMR data were recorded on a 600 MHz Bruker Avance II spectrometer, equipped with a z-axis TCI cryogenic probe and all the experiments were performed at 293 K. The spectra were processed using NMRPipe [25] and displayed with SPARKY [26].

### Crystallization, X-ray data collection and structure determination

DRV was obtained through the NIH AIDS Research and Reference Reagent Program, Division of AIDS, NIAID, NIH. The crystals of inhibitor-free PR^S17^ and PR^S17^/DRV were grown by the hanging drop vapor diffusion technique at room temperature. The crystals were obtained by mixing 1 μl of PR^S17^ or PR^S17^/DRV complex at 5 mg/ml and 1 μl of reservoir solution. In the absence of inhibitor, PR^S17^ crystals grew from a mother liquor containing 2.1 M sodium chloride and 0.1 M HEPES (*4-(2-hydroxyethyl)-1-piperazineethanesulfonic acid*) buffer at pH 7.6. For PR^S17^/DRV complex, DRV was mixed with PR^S17^ at a 5:1 molar ratio and incubated on ice for 30 minutes prior to crystallization trials. The well solution for crystal growth contained 35% Tacsimate^™^, pH 7.0 (Hampton Research Corp., Aliso Viejo, CA). Tacsimate contains 1.83 M malonic acid, 0.25 M ammonium citrate tribasic, 0.12 M succinic acid, 0.3 M DL-malic acid, 0.4 M sodium acetate trihydrate, 0.5 M sodium formate, and 0.16 M ammonium tartrate dibasic. The crystals were cryo-cooled with a mixture of mother liquor and 30% glycerol. Diffraction data were collected at 100° K on beamline 22-ID (SER-CAT) at the Advanced Photon Source, Argonne National Laboratory (Argonne, Il, USA). Data were integrated and scaled with HKL2000 [27].

Structures of PR^S17^, both with and without bound DRV, were solved by molecular replacement using Phaser [28–29]. PR^S17^/DRV complex crystals belong to P6_1_ space group with a dimer in asymmetric unit. The dimer from a knownPR20/DRV complex structure (3UCB) was used as the starting model to solve the structure of PR^S17^/DRV complex. The PR^S17^ mutations were incorporated in the model based on the primary sequence and difference density maps. Inhibitor-free PR^S17^ crystallized in P3_2_21 with a monomer in the asymmetric unit. A monomer of PR20 open form with yttrium bound at the active site (3UF3) was used as the initial model after deleting the flap (residues 43–58). Mutations of PR^S17^ and the deleted flap residues were introduced during the refinement. The atomic models were refined by iterative rounds of model building into electron density maps and refinement using COOT [30] and REFMAC [31]. The surface loops with ambiguities were pruned during early stages of refinement and successfully rebuilt. Two alternate orientations of DRV were fitted into unambiguous electron densities in the PR^S17^/DRV complex. Solvent molecules were inserted at stereochemically reasonable positions using 2Fo-Fc and Fo-Fc maps contoured at 1 and 3 sigma levels, respectively. Structure Figures were prepared with PyMOL (http://www.pymol.org).

## Results and Discussion

### Overall structure

**Fig 1:**
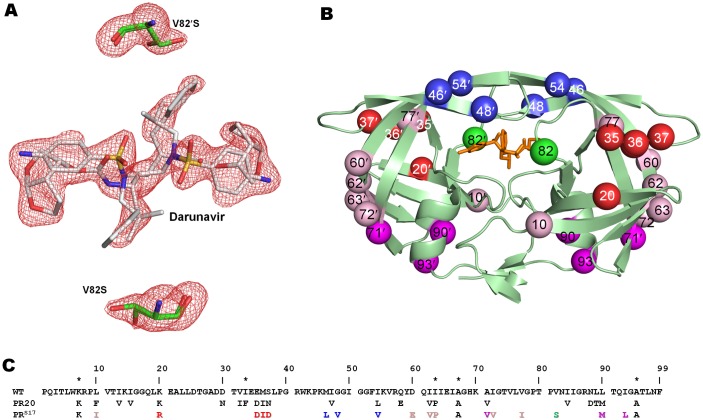
Two Alternate Conformations of Darunavir and the Active Site Mutation V82S in PR^S17^/DRV Dimer. A. F_o_-F_c_ omit map contoured at 3σ level shows Ser82 and DRV have two alternate conformations in both subunits. B. Sites of 17 mutations are mapped on PR^S17^ (pale green cartoon representation) with bound DRV shown as orange sticks. V82S mutation, proximal to the active site, is shown as a green sphere in each subunit. The mutations in the hinge loop cluster are colored as red spheres while the flap mutation cluster is represented as blue spheres. Note that K20R interacts with residues in the hinge loop although it is not contiguous in sequence with this region as described later. The distal mutations perturbing active site aspartates are colored as magenta spheres and remaining mutations are shown as pink spheres. C. Sequence alignment of PR^S17^ with PR and PR20. Mutations introduced in WT PR to restrict autoproteolysis (Q7K, L33I and L63I) and avoid cysteine-thiol oxidation (C67A and C95A) are indicted by asterisks. Residues identical to PR in PR20 and PR^S17^ are omitted. The 17 mutations of PR^S17^ are colored similar to 1B.

PR^S17^ was crystallized in the inhibitor-free form and in complex with DRV. The data collection and refinement statistics are summarized in Table 1. In the absence of inhibitor, PR^S17^ crystallized in the trigonal space group of P3_2_21 with one monomer per asymmetric unit. The residues are numbered 1–99. The PR^S17^/DRV complex crystallized in hexagonal P6_1_ space group with a dimer of subunits numbered 1–99 and 1′-99′ in the asymmetric unit. Both crystals diffracted to 1.5 Å resolution and were refined to R-factors of 13.8 and 14.2 for inhibitor-free PR^S17^ and PR^S17^/DRV complex, respectively. The 17 mutations in PR^S17^ and PR^S17^/DRV complex were unambiguously modeled in the electron density maps (Fig 1A). Interestingly, only two mutations of PR^S17^, G48V in the flaps and V82S in the 80’s loop, have contacts with DRV, while the other 15 mutations alter residues outside the active site cavity (Fig 1B). Sequences of PR, PR20 and PR^S17^ are shown in Fig 1C.

**Table 1:** Crystallographic Data Collection and Refinement Statistics.

	PRS	PRS/DRV
Space group	P3_2_21	P6_1_
Cell Dimensions		
a (Å)	49.90	62.85
b (Å)	49.90	62.85
c (Å)	86.63	82.96
γ (°)	120	120
Resolution range (Å)	50.0–1.5	50.0–1.5
Unique reflections	20429	29451
Redundancy	5.6 (3.8)	5.7 (5.0)
Completeness	99.5 (99.5)^a^	98.9 (97.8)
<I/σ(I)>	25.2 (3.1)	24.8 (4.2)
R_sym_ (%)	5.7 (44.7)	5.7 (40.9)
Refinement resolution range (Å)	50–1.5	50.0–1.5
R_cryst_ (%)	13.8	14.2
R_free_ (%)	18.6	19.6
Number of solvent molecules	130	90
Average B-factor (Å^2^)		
Main chain	23.0	31.9
Side chain	27.8	38.9
Inhibitor		24.4
Solvent	37.3	41.4
RMS deviations from ideality		
Bond length (Å)	0.03	0.03
Angles (°)	2.6	2.8

^a^ Values in parentheses are given for the highest resolution shell (1.55–1.5 Å)

The inhibitor-free structure has 9 residues showing alternate conformations while the PR^S17^/DRV complex has 14 alternate conformations in subunit A and 8 in subunit B. Interestingly, 6 of the residues with alternate conformations in subunit A of the PR^S17^/DRV complex are in the flap region, while subunit B and the inhibitor-free form each have a single residue with alternate conformation in their flaps.

The PR^S17^/DRV dimer superposes on the wild type PR/DRV structure with a root mean square deviation (RMSD) of 0.79 Å for 198 equivalent Cα atoms. The maximum deviation of 4.0 Å occurs at Asp35. The two monomers of PR^S17^/DRV are more similar to the corresponding monomers of PR/DRV with RMSD values of 0.69 and 0.71 Å for 99 equivalent Cα atoms in subunits A and B, respectively. The maximum deviation of 3.8 Å occurs at both Asp35 and Asp35′ as well. Apart from the hinge loop (residues 34–42), structural deviations from the wild type structure are apparent for the loop residues 63–72 and 14–20, suggesting an effect of the mutations on the conformation of these loops. Despite the presence of a large number of mutations, PR^S17^ shares only one mutation (L90M) and a similar substitution (I54L vs I54V) with PR20. The dimers of PR^S17^/DRV and PR20/DRV superimpose with RMSD of 0.88 Å for 198 equivalent Cα atoms and the maximum deviation of 2.9 Å occurs for the 10’s loop residue Gly17. When the two monomers of PR^S17^/DRV are superimposed with those of PR20/DRV, monomer A shows an RMSD of 0.88 Å with the largest deviation of 2.7 Å at Gly17. Monomer B, however, superposes with the lower RMSD of 0.51 Å for 99 Cα atoms and the maximum deviation of 2.4 Å occurs at Asp35′.

The inhibitor-free structures of PR^S17^, PR20 and wild type PR exhibit more differences than do the corresponding DRV-bound dimers. The 99 Cα atoms of PR^S17^ superpose with the corresponding atoms of wild type PR with a RMSD of 1.48 Å and the maximum deviation of 7.7 Å occurs at the flap tip residue Ile50. Similarly, the Cα atoms of PR^S17^ can be superposed with the two monomers of PR20 with RMSD values of 1.21 and 1.39 Å with the maximum deviation of 5.5 and 6.85 Å occurring again at flap tip residues Gly49 and Ile50 in the two subunits.

### Mutations in PR^S17^ do not alter the inhibitor binding site

**Fig 2:**
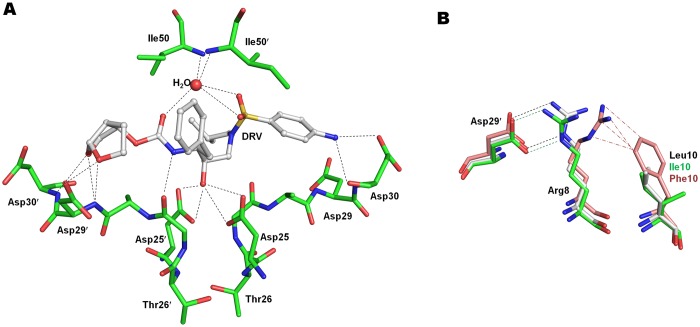
Hydrogen Bond Interactions of Darunavir with PR^S17^ and Effect of Leu10 Mutations. A. Hydrogen bond interactions of DRV with PR^S17^. PR^S17^ is in stick representation with green carbons while DRV is shown in ball and stick with white carbons. For the sake of clarity only one conformation of DRV and Ile50 of PR^S17^/DRV are shown. The hydrogen bond interactions of the second DRV are essentially identical. The hydrogen bonds are shown as broken lines. B. L10I mutation in PR^S17^ does not break the inter-monomer ion pair between Arg8 and Asp29′ unlike L10F in PR20. Wild-type PR is shown with grey carbons, PR^S17^ as green carbons and PR20 as salmon carbons. The van der Waals contacts are represented by (-·-) line. The minor conformation of Arg8 in wild-type PR and PR20 and its interactions with Asp29′ are omitted for clarity.

PR^S17^/DRV complex exhibits the closed flap conformation. As seen in many PR-inhibitor complexes, DRV binds in the active site cavity in two alternate orientations with equivalent occupancy and related by 180°. DRV was designed to form hydrogen bonds with the main chain atoms of protease for tighter binding and thus avoid the detrimental effects of drug resistance mutations at the active site on binding affinity of inhibitors [32]. All hydrogen bond interactions observed between the wild-type PR and DRV are conserved in PR^S17^/DRV (Fig 2A).

The water mediated hydrogen bonds connecting the flap residues Ile50 and Ile50′ to the P2 carbamate carbonyl and the P2’ sulfonamide of DRV are also preserved in PR^S17^/DRV complex. The central transition state mimetic hydroxyl group of DRV forms strong interactions with the carboxylate groups of Asp25 and Asp25’ in the catalytic triad. The conformation of the catalytic triad residues (Asp25, Thr26 and Gly27) is highly conserved in the inhibitor complexes and inhibitor-free PR^S17^ and PR structures.

In PR20, four (D30N, V32I, I47V and I84V) of the 19 mutations alter residues in the active site cavity. These mutations contribute to the S2/S2′ binding pocket and alter the charge, shape and expand the size of the pocket, thereby decreasing the affinity for inhibitor. Unlike PR20, PR^S17^ has only one mutation V82S in the active site cavity. In PR^S17^/DRV complex, the side chain of V82S forms van der Waals contacts with P1 phenyl and P1′ isobutyl in the two subunits similar to the interactions of Val82 in wild type PR/DRV complex. However, the main chain atoms of 80’s loop from Thr80 to V82S shift by ~ 0.8 Å in both the subunits of PR^S17^/DRV in comparison to PR/DRV. This shift enables the Cβ of mutated Ser82 in PR^S17^ to retain van der Waals contacts observed between the Cγ of Val82 and inhibitor in the wild type PR complex. This structural shift is consistent with those reported for V82A single mutant complexes with DRV, indinavir and SQV, which enable the mutant PR to maintain contacts with inhibitors [33–35]. The active site environment and inhibitor/substrate binding cavity are very similar in PR^S17^ and wild-type PR. PR^S17^ exhibits ~3 fold more favorable binding affinity (*K*_m_) for a chromogenic substrate relative to PR20 [17], which is consistent with its binding pocket being more similar to wild-type PR than seen for PR20. However, both PR^S17^ and PR20 have similar catalytic efficiency (*k*_cat_/*K*_m_) as the *k*_cat_ of PR20 is twice that of PR^S17^. This suggests a difference between the transition states (as contrasted with substrate binding) for substrate cleavage by the two enzymes.

### L10I mutation does not alter inter-monomer ion pair

In several drug resistant variants, accessory mutations at Leu10 are observed for all clinical drugs except DRV [5]. Mutation L10I was shown to decrease viral replication and confer resistance to SQV [36]. In PR20, L10F mutation acts to break the inter-subunit ion pair between conserved Arg8 and Asp29′ by shifting the side chain of Arg8 into a new conformation that makes van der Waals contacts with Phe10 [12–13] (Fig 2B). Both Arg8 and Asp29′ make critical interactions with inhibitors and contribute to the S2/S2′ pockets in PR dimers. Loss of this ion pair produces a temperature sensitive phenotype with altered catalytic activity and thermal stability [37–38]. In PR^S17^/DRV complex, however, L10I mutation does not alter the conformation of Arg8 and the ion pair between Arg8/8′ and Asp29′/29 is intact for both the subunits as seen for wild type PR (Fig 2B). In addition, the inhibitor-free PR^S17^ structure shows no conformational change in Arg8 due to L10I mutation. This structural feature is consistent with a strict linear relationship between protein concentration and catalytic activity for PR^S17^ with the very low dimer dissociation of <10 nM comparable to that of wild type PR[17]. Thus, L10I mutation in PR^S17^ does not break the inter-subunit ion pair, unlike the L10F mutation in PR20, and hence does not alter the shape of S2/S2′ pocket or the dimer stability of PR^S17^.

### Hinge loop mutations alter flaps of PR^S17^

**Fig 3:**
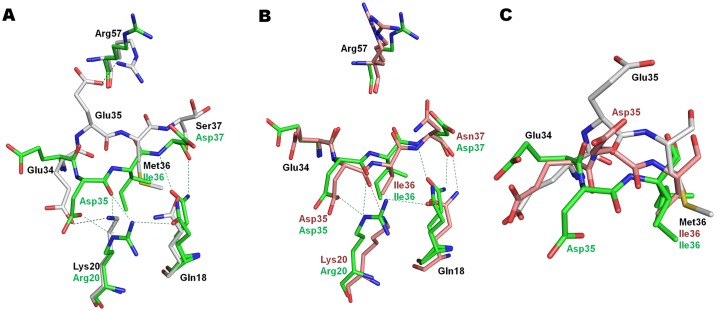
Conformational Changes in Hinge Loop Mutations E35D, M36I and S37N. A. Conformational changes in the hinge loop of PR^S17^/DRV complex in comparison to PR/DRV. B. Comparison of hinge loop between PR^S17^/DRV and PR20/DRV. C. Hinge loop conformation of PR^S17^/DRV, PR/DRV and PR20/DRV in subunit B. Wild-type PR, PR^S17^ and PR20 carbons are shown in grey, green and salmon, respectively.

Comparison of the DRV-bound complexes of PR^S17^ and wild type PR/DRV shows a large conformational change in the hinge loop region (residues 34–42) associated with mutations E35D, M36I and S37N in PR^S17^ (Fig 3A).

In PR^S17^/DRV, mutation to a smaller side chain for E35D leads to loss of the ion pair observed in wild type PR between the side chains of Glu35 and flap residue Arg57. The main chain atoms of Asp35 in PR^S17^/DRV structure trace a direction different from those of Glu35 in PR/DRV. The RMSD of 4.05Å for residue 35 is the largest between the two structures. The orientation of the two side chains, Glu35 and Asp35, differs by ~180°. The new conformation of Asp35 in PR^S17^/DRV is anchored by formation of an ion pair with the Arg side chain of K20R. The guanidine head of K20R also forms hydrogen bond interactions with the main chain carbonyl oxygen of E35D. In addition, the adjacent Met36 side chain interacts with Ile15 and Ile33 in wild-type structures, but the M36I mutation in PR^S17^/DRV alters the main chain (Cα RMSD of 2.3 Å) such that the shorter Ile36 side chain retains the interactions with Ile15 and I33L in PR^S17^. Though the side chain of neighboring S37D mutation in PR^S17^/DRV has no contacts with other PR^S17^ residues, the main chain conformation is rearranged (Cα RSMD 1.7 Å) so that the main chain amide and carbonyl oxygen atoms form hydrogen bonds with one of the alternate side chain conformations of Gln18. Also, I62V mutation in PR^S17^ results in loss of van der Waals contact with Pro39 further altering the hinge loop in comparison to PR/DRV. This conformational change of the hinge loop in PR^S17^/DRV due to K20R, E35D, M36I and S37D mutations is similar in both subunits. Comparison of PR^S17^/DRV with PR20/DRV complex, which has E35D, M36I, S37N and I62V mutations, reveals that the hinge loop conformation of PR20 in subunit A is similar to PR^S17^ except for absence of the K20R mutation and consequent absence of the ion pair interactions observed between Arg20 and Asp35 in PR^S17^/DRV (Fig 3B). In subunit B, the conformation of E35D in PR20/DRV is nearly in-between those of corresponding residues in PR/DRV and PR^S17^/DRV with a RMSD of 2.6 Å between the Cα of PR20/DRV and PR^S17^/DRV (Fig 3C). Like in subunit A, E35D in subunit B of PR20/DRV does not form an ion pair with Lys20. Molecular dynamics studies predict increased flexibility of the flaps due to rearrangement of hinge loop induced by mutations E35D and M36I [39–40]. PR20, which has the similar twisting of hinge loop as in PR^S17^, was shown to remain in open form for longer periods of time than wild-type PR, leading to weaker inhibitor binding at the active site [41]. In addition, PR20 shows altered flap dynamics and the two flaps tend to fluctuate independently of each other unlike in wild-type PR [42]. Thus, E35D, M36I and S37D mutations in PR^S17^ twist the hinge loop thereby breaking the ion pair anchor to the flaps. This rearrangement is expected to increase the flexibility of the flaps of PR^S17^ similar to PR20. In this regard, effects modulated by the flap dynamics and increased flexibility in PR^S17^ may enhance its ability to dissociate from inhibitors.

### Curling of flaps by mutations M46L, G48V and I54V

**Fig 4:**
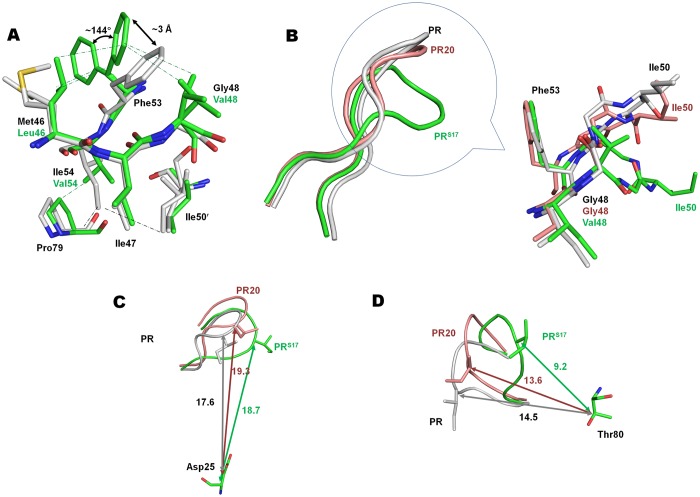
Conformational Changes induced by Flap Mutation Cluster of M46L, G48V and I54V. A. Flap mutation cluster of M46L, G48V and I54V in PR^S17^/DRV compared to PR/DRV. B. Curling of flap due to flap mutation cluster in inhibitor-free structures of PR^S17^ (green ribbon), PR (grey ribbon) and PR20 (salmon ribbon). The conformation of flap tip in the circled portion is shown in sticks for inhibitor-free PR^S17^ (green carbon), PR (grey carbon) and PR20 (salmon carbon). C. Distance in (Å) between the flap tip residue Ile51 and the catalytic Asp25 in inhibitor-free PR^S17^, PR and PR20. D. Distance (Å) between the flap tip residue Ile51 and Thr80 in the three inhibitor-free structures.

The flaps of PR^S17^ harbor 3 mutations, M46L, G48V and I54V. M46L is a major mutation for indinavir resistance and also occurs as minor mutation for all other clinical PIs, except SQV and DRV [5]. SQV selects for G48V as a major resistance mutation. G48V is also a minor mutation for atazanavir. Similarly, I54V is an accessory mutation for all clinical drugs except DRV and nelfinavir [5]. Clearly, flap mutations in the protease can impart cross resistance to PIs. Conformational changes associated with mutation cluster of M46L, G48V and I54V are illustrated in Fig 4.

In comparison with PR/DRV complex, substitution of a larger valine side chain, G48V, in PR^S17^/DRV complex is associated with two alternate conformations of the Phe53 side chain (Fig 4A). Although only two conformations were modeled, the side chain of major conformation of Phe53 exhibits additional conformations in both subunits. In both subunits of PR^S17^, the minor conformation of Phe53, which is the only conformation seen in wild-type PR, is displaced ~3 Å by the mutated Val48 side chain in comparison to the wild-type structure. The major conformation of Phe53 is rotated by ~144° in subunit A and ~170° in subunit B, about the Cα-Cβ chi1 angle. Val48 side chain has van der Waals contact with only the minor conformation of Phe53 in subunit A. In PR^S17^/DRV complex, M46L side chain has van der Waals contact with both alternate conformations of Phe53. I54V mutation of PR^S17^/DRV retains van der Waals contacts with the 80’s loop as observed in PR/DRV, but loses contacts with the side chain of Ile50′ in subunit A. However, in subunit B interactions are retained with both Ile50 and the 80’s loop residues. Thus, M46L and G48V mutations in PR^S17^/DRV synergistically alter the Phe53 conformation by steric hindrance. In PR20/DRV complex, which lacks M46L and G48V mutations, the side chain of Phe53 shows a similar conformation to that in PR/DRV complex.

Different interactions occur in the inhibitor-free PR^S17^ structure. The Phe53 side chain has a single conformation and lacks van der Waals contacts with the side chains of M46L or G48V. Comparison of the inhibitor-free PR^S17^ structure with wild-type PR open form [43] (2PC0) shows that the tip of the flaps in PR^S17^ is twisted from residues G48V to Gly52 (Fig 4B). This twist is initiated by ~176 degree change in φ angle of G48V compared to Gly48 in wild-type PR. The open flaps of PR20 structure [13] (3UF3) do not show this twisted flap tip, but the open conformation flap of F53L single mutant structure [44] (2G69) has a similar twist extending from G48 to F53L. The recently reported protease, FS5929R (5B18), generated by *in vitro* selection has >175-fold increased resistance to DRV and the structure shows a unique twist in the flap conformation [45]. This inactive protease with active site D25N mutation has 22 other mutations (L10I, V11I, L23I, V32I, L33F, S37N, M46I, I47V, I50V, F53L, I54V, Q58E, D60E, L63P, H69R, A71V, G73S, V77I, V82F, L89V, L90M, I93L) including 6 DRV resistance mutations (V11I, V32I, L33F, I47V, I50V, L89V). The flap in FS5929R crystal structure shows marked deviations from the wild-type flap conformation from Gly48 to F53L. Ile50 at the tip of the flap of inhibitor-free PR^S17^ has the largest Cα RMSD of 7.6 Å compared to wild-type PR [43] and deviates by 5.1 and 6.8 Å for subunits A and B of PR20 [13], respectively. Ile50 of PR^S17^ shows lower differences of 3.3 Å and 4.8 Å when compared with the open forms of F53L single mutant [44] and FS5929R [45], respectively. The changes in flap conformation can be described by two components: a vertical component which can be quantified by the distance of flap tips from the catalytic Asp25 and a lateral component which can be defined as the distance between the flap tip and the 80’s loop. PR20 has a wide open flap with a separation of 19.3 Å between Ile50 Cα and the Cα of catalytic Asp25. The corresponding value for PR^S17^ is 18.7 Å and shorter distances of 17.6, 16.2 and 14.9 Å are seen for wild-type PR, F53L single mutant and FS5929R, respectively (Fig 4C). For the lateral component, the distance between the Cα of Ile50 and Thr80 of PR^S17^ is 9.2 Å (Fig 4D). The equivalent values for the wild-type, PR20 and F53L mutant are 14.5, 13.6, and 8.3 Å, respectively. The unique twist at the flap tip of FS5929R results in the shortest distance of 7.9 Å between Gly51 and Thr80. This curling of flaps toward the 80’s loop in FS5929R may contribute to its high levels of resistance to DRV and amprenavir [45]. Thus the flap mutation cluster of M46L, G48V and I54V alters the conformation of Phe53 and twists the flap tip such that PR^S17^ has a flap that is nearly open like PR20 but also closer to 80’s loop similar to F53L mutant and highly DRV resistant FS5929R.

### Distal mutations A71V, L90M and I93L perturb the catalytic aspartates

**Fig 5:**
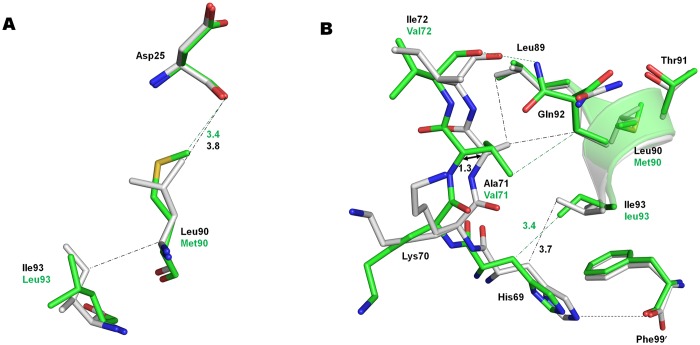
Effects of Distal mutations A71V, L90M and I93L. A. L90M mutation in PR^S17^ (green) induces shortened C-H…O interaction between Met90 and catalytic Asp25 in comparison to wild-type PR (gray). I93L mutation in PR^S17^ results in loss of van der Waals contact observed between Ile93 and Leu 90 of wild-type PR. B. Distal mutations A71V and I93L in PR^S17^ are associated with shift in 70’s β strand by ~1 Å and loss of the ion pair between His69 and the carboxylate tail of the second subunit.

Structural changes associated with neighboring mutations A71V, L90M and I93L are shown in Fig 5.

The L90M mutation in PR^S17^/DRV lies near the main chains of catalytic Asp25/25′ and the longer Met90 side chain forms C-H…O interactions with carbonyl oxygen of Asp25/25′ that cannot form with the shorter Leu90 in wild type enzyme (Fig 5A). The perturbation of the catalytic site is likely to affect inhibitor/substrate binding and catalysis. This interaction between L90M and Asp25/25′ is consistent with those observed for L90M single mutant and also L90M in PR20 [13, 46]. L90M side chains have identical conformations in PR^S17^/DRV and PR20/DRV complexes. L90M single mutants were shown to possess decreased dimer stability [35] and altered catalytic activity [46–47]. Even with no direct contact to inhibitors, L90M induces cross resistance to all clinical protease inhibitors except DRV and tipranavir [5]. The inhibitor-free and DRV complexes of wild-type PR and PR20 show hydrophobic interactions between the L90/M90 Cα and Ile93, which are absent in both PR^S17^ structures due to I93L mutation. I93L in PR^S17^ is likely to further alter L90M mediated perturbation of catalytic aspartates.

The Ala71 side chain in PR structures protrudes into the groove of the small helix formed by residues Arg87 to Leu93, including Leu90 at the base of catalytic Asp25 (Fig 5B). Ala71 Cβ forms van der Waals contacts with Leu89 and Gln92. The A71V mutation in PR^S17^ introduces a bulky valine side chain that cannot fit in the helix groove, which results in the displacement of A71V Cα by 1.3 and 1.1 Å in the two subunits of PR^S17^/DRV in comparison to PR/DRV. Similar displacement of A71V residue is observed in A71V, V82T and I84V triple mutant structures and was proposed to propagate the shifts through the four stranded β-sheet (residues 24–71) to the catalytic aspartates resulting in altered activity [48]. A71V in PR^S17^/DRV loses van der Waals contact with Leu89 but retains interaction with Gln92 in comparison to PR/DRV. The displacement induced by A71V mutation in PR^S17^ affects adjacent residues His69 and I72V since the Cα atoms of these residues have shifted by more than 1 Å compared with the positions in PR/DRV. The shift, in addition to steric hindrance by I93L mutation, results in loss of ion pair interaction between the side chain of His69 and the charged carboxylate terminus of the other subunit in PR^S17^/DRV. PR20 bearing both L90M and A71V mutations exhibits a similar shift in Cα of A71V. However, His69 of PR20/DRV retains the ion pair with the carboxylate terminus as in PR/DRV due to absence of I93L mutation. His69 in PR plays a critical role in autoprocessing, and mutation H69E that introduces a negative charge next to the carboxylate end was shown to impede folding and maturation [49]. However, PR ^S17^ undergoes efficient autoprocessing similar to wild-type PR [17]. A71V and L90M together are considered as resistance mutations for all clinical drugs except DRV and tipranavir [5]. In addition to A71V and L90M, I93L is selected as a resistance mutation for atazanavir. A71V, L90M and I93L distal mutations likely propagate alterations to the catalytic site thereby inducing cross resistance to different inhibitors.

### Comparison of PR^S17^ mutations with single mutant structures

Several of the mutations in PR^S17^ have been studied in PR with single mutations. Single mutation M46L in PR was shown to increase the *K*_*i*_ value for DRV by 10-fold [50]. However, comparison of structures shows that the conformation of M46L mutation in PR^S17^/DRV complex differs from that observed in the DRV complex with M46L single mutant (PDB id: 2HS2) [50]. In addition, the Cα atoms of M46L in PR^S17^/DRV dimer are shifted by ~ 0.7–1.0 Å compared to its position in single mutant structure. In PR^S17^/DRV, the side chain conformation of M46L is influenced by the altered conformation of Phe53 due to the neighboring G48V mutation, as explained earlier. The single mutation of G48V in the flap results in 29-fold weaker *K*_*i*_ for DRV. Though the conformation of the Val48 side chain is similar in PR^S17^/DRV and the G48V single mutant complex with DRV (PDB id: CYW) [51], the main chain of PR^S17^ shows shifts of ~1 Å at G48V (subunit A) or ~0.7 Å at Phe53 and M46L (subunit B) further indicating that these residues act synergistically. The conformation of I54V mutation in PR^S17^/DRV also differs from that observed in the single mutant complex with DRV (PDB id: 3D20) [51] that showed 8-fold increase in the *K*_*i*_ for DRV. The Cα atoms of mutated I54V are also shifted by ~0.6–1.3 Å in the two subunits of PR^S17^/DRV. No interaction was observed between I54V and 80’s loop in the single mutant complex, while I54V in PR^S17^ forms van der Waals and C-H…O interactions with Pro79 of 80’s loop. As reported in the previous section, the L90M mutation forms shortened C-H…O interaction (3.1–3.4 Å) between the Met90 side chain and the main chain carbonyl of Asp25 in PR^S17^/DRV complex instead of longer van der Waals interaction (3.7–3.8 Å) observed in PR/DRV complex. The L90M single mutant complex with DRV exhibited a similar shortened interaction with Asp25 and decreased the PR dimer stability [46]. These comparisons emphasize that the conformation of a mutated residue is influenced by its neighboring residues and may not be similar to those observed in single mutant structure. Taken together, comparison of PR^S17^/DRV complex with the single mutant complexes indicate that the effects seen in PR^S17^ are not a simple addition of single mutations, but synergistic effects of clusters of mutations that can propagate from distal regions.

### Solution conformation of inhibitor-free PR^S17^ studied by NMR

**Fig 6:**
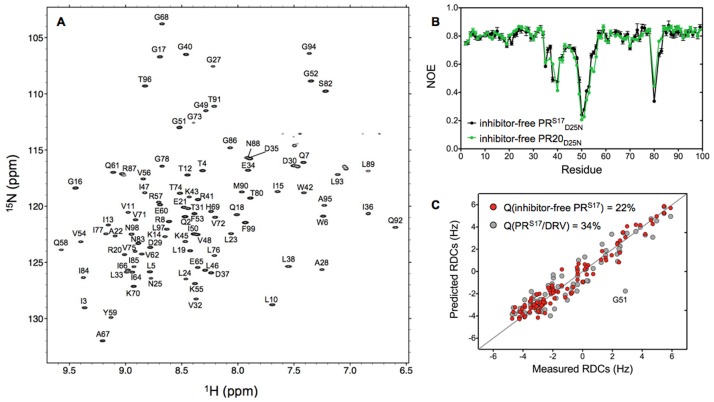
NMR Analysis of Solution Conformation of Inhibitor-free PR^S17^_D25N_. A. ^1^H-^15^N TROSY-HSQC spectrum of the inhibitor-free PR^S17^_D25N_ recorded in 20 mM sodium phosphate pH 5.7 at 20°C. B. ^15^N NOE measured under the same conditions for the inhibitor-free PR^S17^_D25N_ (black) and PR20_D25N_ (green) as a function of the residue number. C. Comparison between the ^1^D_NH_ RDCs measured for the inhibitor-free PR^S17^_D25N_ in a dilute solution of squalamine with those predicted from the crystal structures of the inhibitor-free PR^S17^ (red dots) and PR^S17^/DRV (gray dots). All the NMR experiments were recorded at 600 MHz.

To gain insight into the conformational dynamics of the inhibitor-free PR^S17^, we investigated the inactive variant PR^S17^_D25N_ by NMR spectroscopy. The ^1^H-^15^N TROSY-HSQC spectrum recorded at 20°C and pH 5.7 shows the typical well-resolved and dispersed amide resonances characteristic of a folded protein (Fig 6A). Using a standard combination of triple resonance experiments, HNCO and HNCACB, we fully assigned the backbone chemical shifts of the inhibitor-free PR^S17^_D25N_ (Fig 6A).

Previous ^15^N relaxation measurements have shown that the flaps of the inhibitor-free protease are flexible over a wide dynamic range, from sub-ns to ms timescale [7, 23]. The ^15^N NOE data measured here at 600 MHz confirmed that the flaps of the inhibitor-free PR^S17^_D25N_ are also flexible in solution on a sub-ns timescale (Fig 6B). Comparison of the ^15^N NOE measured for the inhibitor-free PR^S17^_D25N_ and PR20_D25N_ shows very similar profiles with small NOE values for flap residues 48 to 54 and the hinge regions (residues 35 to 40, and 80), indicating that despite small structural differences in the flap conformations these two variants experience similar conformational flexibility in solution (Fig 6B).

We finally determined the preferred flap orientations of the inhibitor-free PR^S17^_D25N_ by measuring RDCs for the backbone amide N-H vectors, using a dilute solution of squalamine to induce a weak alignment of the NMR sample [52]. These RDCs can be measured by imposing a very slight deviation from the random, isotropic distribution of macromolecules in an NMR sample and are very sensitive reporters on the time-averaged orientation of the corresponding inter-nuclear vectors [53]. Although amide RDCs alone are typically insufficient to build a protein structure *de novo*, they are exceptionally well suited to evaluate agreement between the actual state of the protein in solution and the coordinates seen in different crystal structures, as recently demonstrated in the case of the inhibitor-free PR [16]. The ^1^D_NH_ RDCs measured here for the inhibitor-free PR^S17^_D25N_ show an excellent agreement between the measured RDCs and the best-fitted values obtained for the inhibitor-free PR^S17^ X-ray structure, as reflected in a Q-factor of 22% (Fig 6C). As expected, a significantly poorer correlation was obtained when the measured RDCs were compared to the values predicted for the PR^S17^/DRV crystal structure (Q-factor = 34%), especially for the residues in the flaps as exemplified by G51 (the Q-factors calculated for the residues in the extended flap region[16] is 41% for the PR^S17^/DRV structure compared to 19% for the PR^S17^ crystal structures). It should be noted that while residues in the flaps experience ps-ns dynamics, as demonstrated by the heteronuclear NOE measurements (fig 6B), we found no evidence for relaxation on a longer time scale (i.e. no line broadening), especially for G51 (fig 6A), suggesting that the RDCs measured for the flap residues can confidently be fitted to a single structure. Therefore, these results unambiguously confirm that the inhibitor-free PR^S17^_D25N_ adopts an open flap conformation in solution very similar to that described in the present inhibitor-free PR^S17^ crystal structure.

## Concluding Remarks

In contrast to other highly resistant proteases like PR20, PR^S17^ has none of the major mutations (I47V, I50V, I54ML, L76V and I84V) associated with DRV resistance, although it exhibits 10,000-fold weaker binding affinity for DRV relative to the wild type PR. This suggests that the molecular basis of drug resistance may differ in PR^S17^ and PR20. PR^S17^ mainly differs from the wild-type PR by having clusters of mutations that lack direct interactions with the inhibitor. Unlike PR20 where the mutations expand the S2 pocket, the mutations in PR^S17^ do not substantially alter the active site cavity. Except for V82S at the active site and flap mutation G48V, all other mutations are distal to the active site, yet PR^S17^ exhibits poor binding affinity for clinical PIs and synergistic changes that alter the conformation and dynamics of the flaps. Therefore, these clusters of mutations give rise to long range and dynamic effects that diminish the binding affinity of PIs. These effects also extend to inhibitors like DRV, which were specifically designed to tolerate mutations in the active site cavity by forming main chain hydrogen bonds with PR, with diminished binding to these mutants.

It has been suggested that selection for the open-flap conformation alters the folding landscape of highly drug resistant mutants, exemplified by PR20, to avoid a free-energy trap of inhibitor-bound enzyme [54]. This all-in-one mechanism is beneficial for viral survival since it is independent of substrate binding at the active site and possibly confers cross resistance to multiple drugs by providing an escape pathway. As the machine learning algorithm selected PR^S17^ as representative of a wide class of highly resistant mutants, we expect this to likely signify a common mechanism of drug resistance. Moreover, PR^S17^ could prove to be an excellent prototype to design inhibitors that overcome drug resistance induced by distal mutations.
